# The use of gauze-based negative-pressure wound therapy with Y-connector for dressing full-circumference skin grafts on both lower limbs

**DOI:** 10.1093/jscr/rjab266

**Published:** 2021-06-29

**Authors:** Michika Fukui, Natsuko Kakudo, Masakatsu Hihara, Toshihito Mitsui, Yuki Matsuoka, Atsuyuki Kuro, Kenji Kusumoto

**Affiliations:** Department of Plastic and Reconstructive Surgery, Kansai Medical University, Osaka, Japan; Department of Plastic and Reconstructive Surgery, Kansai Medical University, Osaka, Japan; Department of Plastic and Reconstructive Surgery, Kansai Medical University, Osaka, Japan; Department of Plastic and Reconstructive Surgery, Kansai Medical University, Osaka, Japan; Department of Plastic and Reconstructive Surgery, Kansai Medical University, Osaka, Japan; Department of Plastic and Reconstructive Surgery, Kansai Medical University, Osaka, Japan; Department of Plastic and Reconstructive Surgery, Kansai Medical University, Osaka, Japan

**Keywords:** gauze-based negative-pressure wound therapy, Y-connector, easy method to dress skin graft

## Abstract

Free skin grafts have long been used as an essential surgical procedure to treat skin defects due to burns, trauma or illness, requiring skin transfers from sufficient, non-marked areas. Usually, free skin grafts are covered by simple pressure dressings or tie-over dressings. However, under conditions of wide, concave or irregular wounds, graft removal has been problematic due to instability. We report a case in which gauze-based negative-pressure wound therapy using a Y-connector was indicated for the opportunity to cover both limbs with free skin grafts all around, providing successful wound care.

## INTRODUCTION

Free skin grafts are often applied to treat various wounds, especially in the field of plastic and reconstructive surgery. In cases where the wound area is large, a meshed skin graft is usually indicated. Various methods are available to dress meshed skin grafts, such as simple pressure dressing, tie-over dressing and negative-pressure dressing, but dressing large, concave or uneven wounds is difficult.

We were able to dress very large, full-circumference, complicated wounds on both limbs with meshed skin grafts by applying gauze-based negative-pressure wound therapy (NPWT) with a Y-connector.

**Figure 1:**
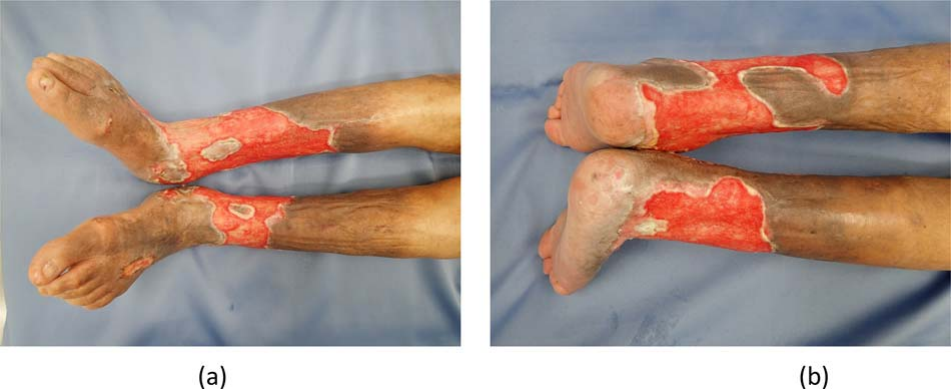
(**a**) Front side of both limbs. (**b**) Back side of both limbs.

## CASE REPORT

A 62-year-old woman with venous ulcers on both lower limbs had been treated in the dermatology department, experiencing repeated exacerbations and remissions over a 10-year period.

She was hospitalized in the dermatology department due to wound infection. The infected wounds were treated with antibiotics and ointments, and granulation tissue formed. She was referred to us for treatment of ulcers on both lower limbs.

**Figure 2:**
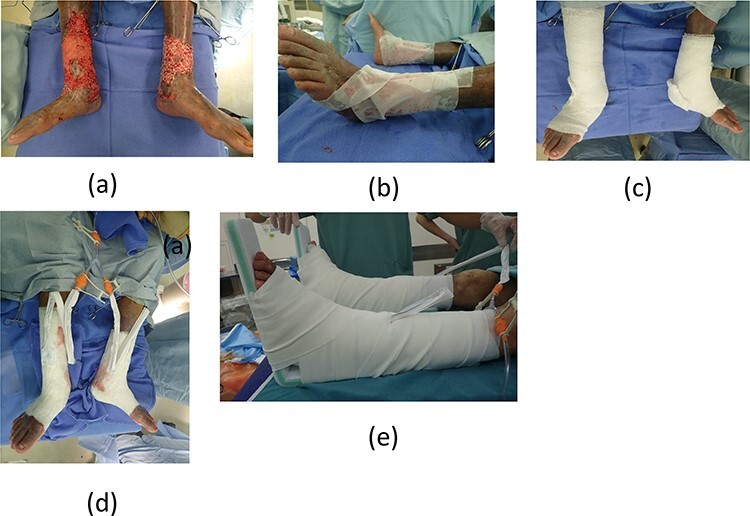
(**a**) Meshed skin grafts fixed to the wound beds. (**b**) Meshed skin grafts were covered with silicon gauze. (**c**) Gauze filler was rolled around several times. (**d**) Covered with waterproof films and attached Y-connector. (**e**) Whole legs were fixed with splints.

**Figure 3:**
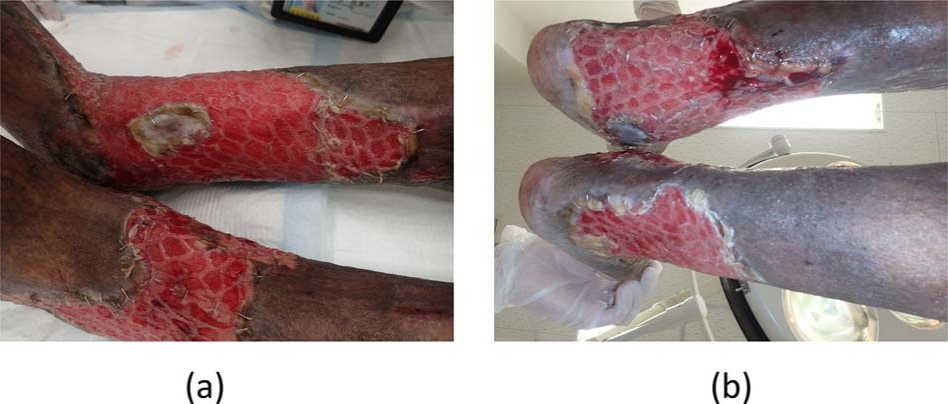
(**a**, **b**) All meshed skin grafts took.

**Figure 4:**
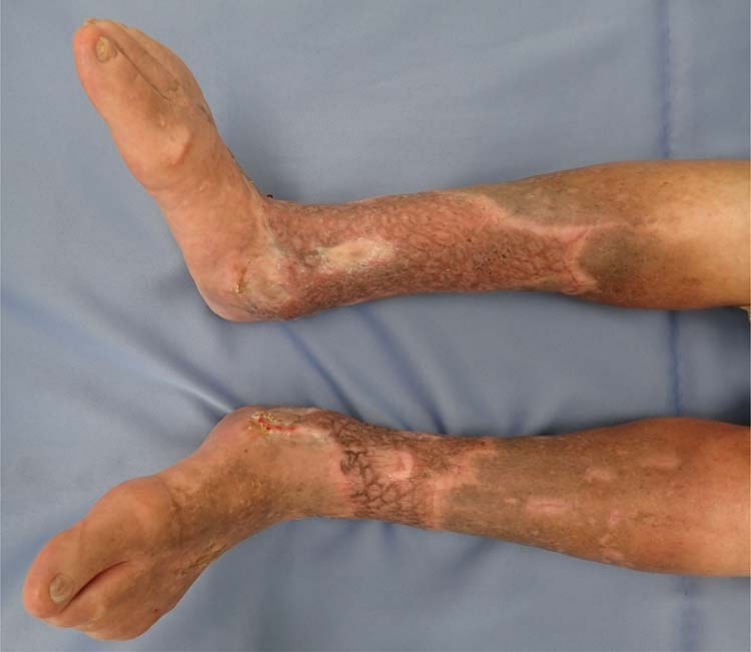
Postoperative 2 months.

The ulcers affected the entire circumference of both limbs (Fig. 1a and b). Wound areas were very large, with complicated shapes. We performed split-thickness meshed skin grafts to treat the wounds under local anesthesia. Graft skin was obtained from the abdomen and processed to a thickness of 0.002 inches and meshed six times. Skin grafts were fixed with surgical staples to the wounds and covered with silicone gauze (Fig. 2a and b). Gauze-based NPWT was used to dress the skin grafts. We covered the silicone gauze with several layers of gauze filler, then a waterproof film (Fig. 2c). Negative pressure was set at 125 mmHg. A Y-connector enabled application of suction to both limbs using a single system (Fig. 2d). Both legs were fixed with splints (Fig. 2e). At 6 days postoperatively, we removed the dressing gauze after wetting with saline solution. The patient experienced no pain while removing the gauze dressing. All meshed skin grafts took successfully (Fig. 3a and b). Figure 4 shows the wounds on both limbs 2 months postoperatively.

## DISCUSSION

Skin graft is one of the most indispensable techniques in plastic surgery and is often used to treat various wounds. A split-thickness meshed skin graft is usually used for large or complicated wounds. However, dressing a large or complicated skin graft is very difficult due to the factors such as the site on the body and size and shape of the wound, such as multiple wounds or those affecting the pudendum, entire circumference of the arm or legs.

The dressing for skin grafts has a greater influence on graft survival than on graft fixation. The main purpose of dressing is to allow the skin graft to rigidly attach to the wound bed [1].

Three main methods can be used to fix skin grafts [1]. The first method is the simple pressure dressing. Silicone gauze is placed on the skin graft, overlaid with gauze, then fixed with tape, rubber bands or surgical staples.

The second method is tie-over dressing. After placing silicone gauze on the skin graft, a wetted piece of raw cotton is placed over the silicone gauze and fixed with nylon thread.

Disadvantages of these conventional methods are the time required to sew with nylon threads and the stitch marks surrounding the skin graft. Moreover, these methods of fixation are difficult depending on the place, size and irregularity of the wound.

The third method is use of a negative-pressure dressing. This method uses NPWT to dress skin grafts. A key advantage is that this method is not time-consuming and can be used on sites where simple pressure or tie-over fixation is difficult. Moreover, the continuous negative-pressure environment provided by NPWT reduces the formation of subcutaneous hematoma. Yin *et al*. [2] reported that NPWT increased the rate of graft take and reduced the rate of reoperation when applied to cover the wound bed with skin graft compared with conventional methods.

NPWT consists of a negative pressure source and a wound filler material. Filler materials are roughly divided into two types: foam filler or gauze filler. Both fillers are used to fix the skin graft. However, gauze filler is more appropriate than foam filler to fix irregularly shaped, complicated and/or large wounds. Folding or changing the shape and covering large areas are easily achieved with gauze filler. In this case, both wounds extended the entire circumference of each limb, making the affected areas very complicated and large. The use of gauze filler enabled us to shorten the operation time due to the ease, application of uniform negative pressure and easy control of exudate. Some papers reported that gauze-based NPWT offers advantages for the surgical team regarding the ease of application to large and/or complicated wounds [3, 4]. The rate of graft take with gauze-based NPWT has been reported as 96% [4]. We consider gauze-based NPWT as the best method to dress skin grafts for full-circumference wounds of the legs, arms or body, given the simplicity and speed of the technique. The effectiveness of gauze-based NPWT for the treatment of large, irregular areas of trauma such as military wounds is suggested [5, 6].

In this case, we were able to apply negative pressure to both limbs together using the Y-connector. A Y-connector enables treatment of two sites at the same time while also reducing medical costs.

Moreover, gauze filler reportedly reduces patient pain associated with removing the gauze [3]. Little force is required to remove gauze filler compared with foam filler, because tissue shows ingrowth into foam filler, but not into gauze filler [7]. Our patient experienced no pain when we removed the gauze filler.

## CONCLUSION

In our experience, gauze-based NPWT with a Y-connector appears very useful to dress large, complicated skin grafts such as full-circumference wounds on both limbs.

## CONFLICT OF INTEREST STATEMENT

None declared.

## FUNDING

None.
